# The actual and anticipated effects of a menthol cigarette ban: a scoping review

**DOI:** 10.1186/s12889-020-09055-z

**Published:** 2020-07-09

**Authors:** Christopher J. Cadham, Luz Maria Sanchez-Romero, Nancy L. Fleischer, Ritesh Mistry, Jana L. Hirschtick, Rafael Meza, David T. Levy

**Affiliations:** 1 Georgetown University-Lombardi Comprehensive Cancer Center, Cancer Prevention and Control Program, 3300 Whitehaven St. NW, Washington, DC, USA; 2 Department of Epidemiology, University of Michigan School of Public Health, 1415 Washington Heights, Ann Arbor, MI USA; 3 Department of Health Behavior and Health Education, University of Michigan School of Public Health, 1415 Washington Heights, Ann Arbor, MI USA

**Keywords:** Menthol cigarettes, Tobacco regulation, Scoping review

## Abstract

**Background:**

The United States (US) Food and Drug Administration (FDA), under the 2009 Family Smoking Prevention and Tobacco Control Act, banned characterizing flavors in cigarettes; however, mentholated tobacco products were exempt. Since 2009, over 20 US jurisdictions and numerous countries around the world have extended this restriction to menthol. Currently, the FDA is reconsidering its position on a nation-wide menthol cigarette ban. However, the effects of such a ban remain unclear. We conducted a scoping review to explore the impact of a menthol cigarette ban on individual behaviors (initiation, cessation, and product switching), sales, and compliance.

**Methods:**

We conducted a search of the international literature using PubMed, EBSCO, and Web of Science (to November 25, 2019). We retrieved articles relevant to the impacts of an implemented or hypothetical menthol ban. We also included studies of flavored tobacco product bans due to their potential relevance in gauging compliance and product substitutability.

**Results:**

The search identified 493 articles, of which 24 were included. Studies examined the effects of implemented menthol bans (*n* = 6), hypothetical menthol bans (*n* = 12) and implemented flavor bans that exclude menthol (*n* = 6). Menthol bans were found to reduce sales and increase smoking cessation with only partial substitution for non-menthol cigarettes. US smokers’ reactions to a hypothetical ban indicate that about 25–64% would attempt to quit smoking and 11–46% would consider switching to other tobacco products, including 15–30% to e-cigarettes. Flavor ban studies indicate reductions in initiation of 6%. Ban compliance was high, but studies indicate that the tobacco industry and retailers have attempted to circumvent their impact via packaging changes and online sales.

**Conclusion:**

Our review finds that extending the US cigarette flavor ban to menthol products would promote smoking cessation and reduce initiation. This evidence supports further action by the FDA towards mentholated tobacco products. However, few studies have been conducted in the vaping era.

## Background

In 2009, the Family Smoking Prevention and Tobacco Control Act granted the United States (US) Food and Drug Administration (FDA) the authority to regulate the manufacture, sale, distribution, and marketing of tobacco products [1]. Under this act, the FDA banned characterizing flavors in cigarettes, citing their appeal to youth and young adults. Notably, this ban exempted mentholated tobacco products. The FDA recently announced its intention to ban menthol in cigarettes [2]. However, they must demonstrate that such a ban would reduce the initiation of and increase the cessation from the use of tobacco products [2, 3].

As an additive in tobacco, menthol has been marketed in the US since the 1920s. Tobacco companies have targeted the marketing of menthol cigarettes to specific demographics (i.e., young, female, and African-American smokers) and manipulated the menthol content to recruit and retain smokers [4]. As a result, the proportion of US smokers using menthol cigarettes is higher among youth (ages 12–17 years) and young adult (ages 18–25 years) smokers (56.7 and 45.0% respectively, vs. 30.5–34.7% among older age groups); among women (39.6% vs. 31.4% among men); and among African Americans (88.5% vs. 25.7% among Caucasians) according to 2004–2010 data from the National Surveys on Drug Use and Health [5]. The population prevalence of menthol smoking has remained constant in recent years, despite declines in non-menthol smoking [6]. With the stable trend in menthol cigarette use [7], there is growing concern that menthol flavoring continues to increase youth smoking initiation and dependence and reduce cessation [8, 9].

The current availability of menthol cigarettes varies widely, both domestically and internationally, as menthol bans have been implemented at the local and country-level. Over 20 local US jurisdictions in California, Minnesota, Illinois, and Massachusetts have implemented a ban on menthol cigarettes since 2017 [10]. These range from comprehensive jurisdiction-wide bans that include all menthol tobacco products and e-cigarettes, such as in Oakland, CA, to bans in specially designated zones (e.g., near schools) with exemptions for some retailers, such as in Chicago, IL [10]. Outside of the US, several Canadian provinces (Alberta, New Brunswick, Nova Scotia, Ontario, Quebec, Prince Edward Island, and Newfoundland and Labrador) implemented menthol bans prior to the 2017 national ban [11, 12]. Brazil banned all flavor additives, including menthol, in all tobacco products in 2012 and Ethiopia followed in 2015 [11]. Turkey banned menthol cigarettes and hand-rolled tobacco in 2019 and the European Union’s menthol ban will begin in 2020 [11].

Although the role of menthol flavoring in cigarette smoking initiation and cessation has been well-documented [8, 9], it is less clear how a menthol ban may impact these same behaviors. A ban may encourage current menthol smokers to transition to non-menthol cigarettes or an alternative tobacco/nicotine product. Furthermore, the response of manufacturers, distributors, and sellers is unclear. The industry may attempt to circumvent the ban through the introduction of similar, but not banned products, such as the introduction of clove cigars following the US flavored cigarette ban [13]. Bans may also result in the rise of a black market for the banned product.

In order to synthesize the current literature on the potential impact of a comprehensive menthol cigarette ban in the US, we conducted a literature review of studies of a ban’s effects on individual behavior, product sales, and industry compliance. We also included studies of bans on other flavors besides menthol, due to their potential relevance in understanding the impact of a menthol ban on compliance and tobacco product substitutability. We explicitly consider the effects of a menthol ban on smoking initiation and cessation, and switching to other nicotine delivery products.

## Methods

We searched PubMed, EBSCO, and Web of Science for articles using the terms “(menthol OR flav*) AND (tobacco OR cigarette*) AND (ban OR restrict*).” The final searches were conducted on November 25, 2019. The search was carried out with no restrictions on location or year of publication. In addition, reviews of menthol cigarette studies by the FDA [9] and Villanti et al. [8], as well as references of selected papers, were inspected for potentially relevant articles.

Eligibility criteria were determined a priori*.* Peer-reviewed studies were included if they empirically considered the effects of an implemented or hypothetical ban on menthol or all flavor tobacco/nicotine products. Abstracts, letters to the editor, and papers written in a language other than English were excluded. Additionally, studies were excluded if they were not specific to a menthol ban or an implemented flavor ban, if they presented an opinion on a menthol or flavor ban, or if they did not report results specific to individual behavior, sales, or compliance. Studies that examined restrictions that exempted specific locations such as at tobacco bars, vape shops, or over 21 establishments were excluded, since the effects of such regulation would not be comparable to a ban on all retailers. Because a range of heterogeneous studies with different methodologies and outcome measures were included, we were not able to employ a standardized quality assessment typical of systematic reviews. In lieu, we conducted a scoping review where we provide the details on the study questions, methods and results. The protocol for this review is not publicly available.

Three of the authors (CJC, LMSR, DTL) conducted the initial abstract review. Any discrepancies were resolved by consensus. The data abstraction sheet was developed by the three authors and tested with an initial sample of articles. Full-text review and data abstraction were conducted independently by two authors, and discrepancies were resolved by a third. Following abstraction, the articles were categorized by type of ban: 1) implemented menthol ban, 2) hypothetical menthol ban, and 3) implemented tobacco flavor bans that exclude menthol. Within each ban category, the studies were ordered by theme: 1) individual behaviors, 2) individual intentions, 3) product sales, and 4) ban compliance.

## Results

**Fig. 1:**
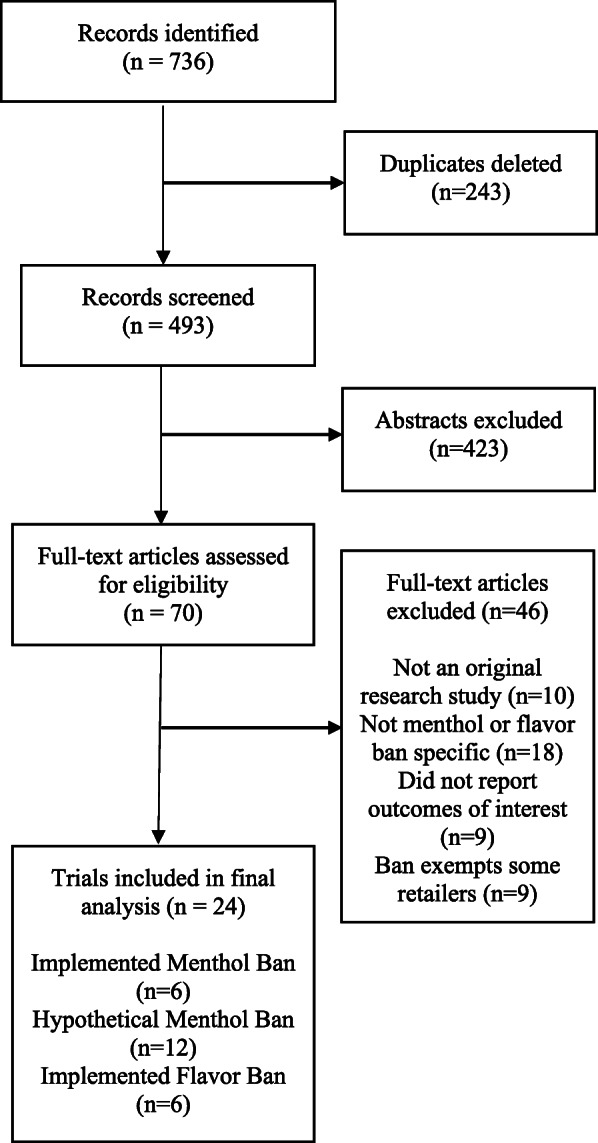
PRISMA Diagram of Identified Studies of Menthol and Flavor Bans

The search identified 493 potentially relevant articles. A total of 70 articles were deemed eligible for full-text review. Only 24 were eligible for inclusion and data abstraction (Fig. 1). Studies were excluded because they were not original research studies (*n* = 10), were not specific to a menthol or flavored tobacco ban (*n* = 18), did not report on the outcomes (behavior, intention, sales and compliance) of interest (*n* = 9), reported outcomes already reported in another study (*n* = 1) or analyzed a ban exempting a large class of retailers, such as tobacco shops or other over 21 establishments (*n* = 9).

Among the 24 included studies, six evaluated the impact of an implemented menthol ban on behavior, sales, or compliance; 12 examined individuals’ behavioral intentions in response to a hypothetical menthol ban; and six assessed the effects of an implemented flavor ban that excluded menthol.

### Implemented menthol ban

Of the six studies that explored the impact of an implemented menthol ban, two evaluated individual behavior, one examined a change in sales, and three assessed compliance (Table 1).

**Table 1:** Studies that report results related to implemented menthol bans

Author, Year	Location	Age group	Study Design (Theme)	Sample Size	Ban Specifics (Implementation Date)	Data Sources	Main Outcomes	Results
Borland, T. 2018 [14]	Ontario, Canada	NA	Pre and post analysis (Compliance)	*N* = 63 packs (*n* = 30menthol and *n* = 33 non-menthol)	2017 Provincial ban on menthol cigarettes and other tobacco products	Eight retail outlets, Toronto, Ontario. Pre-ban Oct/Nov 2016; Post-ban Feb and Sept 2017. Evaluation of menthol packs matched with their non-menthol alternatives	Content analysis of the pack: visual, textual and physical design. Inside and outside colors, foil color, filter tip, filter type, and taste descriptors	Post-ban, blue was the most prominent pack color. Blue and silver filter tips continued to be used. Use of “smooth” instead of “menthol” or “fresh” descriptors in the pack.
Brown, J. 2017 [15]	Alberta and Nova Scotia, Canada	NA	Pre and post analysis (Compliance)	*N* = 498 cigarette packs (*n* = 199 packs of menthols)	2015 Provincial ban on menthol tobacco products (Nova Scotia, May 2015. Alberta, Sept 2015.	Retail outlets cigarette pack purchases. Sept/Dec 2015. Pre-ban in Alberta; Post-ban Alberta and Nova Scotia.	Content analysis of the pack: visual, textual and physical design. In and out colors, foil color, filter tip, filter type, and taste descriptors	Post-ban, no cigarette packs labeled as “menthol” were purchased.Brands identified as menthol pre-ban were repackaged to connote products that were menthol replacements. Menthol was replaced with “Green” descriptor in Alberta.
Chaiton, M. 2019 [16]	Ontario, Canada	NA	Time Series (Sales)	NA	2017 Provincial ban on menthol cigarettes and other tobacco products	Ontario (ban) and British Columbia (no ban) monthly wholesale data from Health Canada from 2012 to 2017 (80 months).	Sales of cigarettes	Post-ban, menthol cigarette sales fell to near zero (55 million unit reduction), a 4% decrease in non-menthol and 11% decrease in total cigarette sales. British Columbia saw a 15% decline in menthol sales, a 1% increase in non-menthol sales and a 1% decrease in overall sales.
Chaiton, M. 2019 [17]	Ontario, Canada	16+	Cohort study (Individual Behavior)	*N* = 913 current smokers	2017 Provincial ban on menthol cigarettes and other tobacco products	Telephone Survey. Baseline Sept/Dec 2016. Follow-up Jan/Aug 2018. Stratified by daily and occasional menthol and non-menthol smokers.	Current use, intention to quit and quit attempt one-year post ban	Post-ban, 56% of all respondents reported at least one quit attempt and 19% reported successful quitting. Menthol users were more likely to have made a quit attempt (62% vs. 43% of non-menthol users). 24% of daily menthol users reported having quit post-ban compared to 19% of non-menthol smokers. Daily and occasional menthol smokers made more quit attempts (adjusted relative rate of 1.62 and 1.09) than non-menthol smokers.
Chaiton, M. 2018 [18]	Ontario, Canada	16+	Cohort study (Individual Behavior)	N = 325 individuals who smoked at least one menthol in the past year	2017 Provincial ban on menthol cigarettes and other tobacco products	Telephone Survey. Baseline Sept/Dec 2016. Follow-up Jan 2018. Stratified by menthol daily, menthol someday and nonmenthol users.	Current use, intention to quit and quit attempt one-month post-ban	Pre-ban, 123 menthol smokers [59.7%] said that they would switch to non-menthol cigarettes, but only 51 (28.2%) switched post-ban.Pre-ban, 30 (14.5%) said they quit vs. 60 [29.1%]) attempted to quit post-ban. Pre-ban, 12 [5.8%] intended to use other flavored tobacco or e-cigarette products (menthol was not banned in e-cigarette products) vs. 60 [29.1%]) who switch post-ban.
Stokolsa, M. 2019 [19]	Nova Scotia, Canada	NA	Time series (Compliance)	NA	2015 Provincial ban on menthol cigarettes	Provincial Tax Commission data on illicit cigarettes seized from 2007/08 to 2017/18. Pre-ban (2012 to 2015). Post-ban (2015–2018) Data not specifically stratified by menthol or non-menthol cigarettes.	Number of seized illicit cigarettes	Post-ban, the number of illicit cigarette cartons decline from > 60,000 to < 10,000 in 2017. Afterward, the seizure volume remained stable, with no statistically significant difference in the number of cigarettes seized before and after the menthol ban. Amount of menthol cigarettes seized was not significant.

#### Individual behavior

Chaiton et al. [17, 18], conducted two studies analyzing the impact of the 2017 Ontario, Canada ban on menthol cigarettes. Both studies used random-digit dialing to identify a sample of smokers aged 16 and older prior to the menthol ban’s implementation. The first study, with 324 smokers at one-month post-ban [18], found that 28% of menthol smokers switched to non-menthol cigarettes, 29% attempted to quit, and 29% used an alternative flavored tobacco or e-cigarette product. A second study [17] conducted one-year post-ban with a sample of 913 smokers (including non-menthol and menthol users) found that 56% of all smokers had made at least one quit attempt, and 19% reported no current tobacco use. Daily menthol smokers were more likely to have made a quit attempt (63% vs. 43%) and quit smoking (24% vs. 14%; adjusted risk ratio 1.62; 95% CI 1.08 to 2.42) compared to daily non-menthol smokers.

#### Sales

Chaiton et al. [16] employed a time series analysis using monthly cigarette wholesale sales data from Health Canada for the years 2012–2017 to examine the effects of Ontario’s menthol ban. They found an overall 11% decline in all cigarette (menthol and non-menthol) sales post-implementation. Immediately after the ban, sales of menthol cigarettes decreased to nearly zero and non-menthol sales fell 4%, but a slight rebound was observed in non-menthol cigarette sales in late 2017. We note that menthol cigarettes accounted for only 5% of the Canadian cigarette market in 2015 [17, 20] and less than 10% of Canadian smokers regularly used menthol cigarettes in 2017 [20], compared to over 30% of US smokers who regularly use menthol cigarettes [21, 22].

#### Compliance

Three studies considered compliance with an implemented menthol ban. Two evaluated changes in marketing practices. Borland et al. [14] used a pre-post analysis to evaluate the change in cigarette packs following a menthol ban in Ontario using a sample of 30 menthol and 33 non-menthol packages. Pre-ban menthol packs were matched with their post-ban non-menthol alternatives. Following the ban, packs did not include menthol descriptors but still used blue as the most common color for the menthol brand replacements packages which no longer contained menthol. Brown et al. [15] conducted a similar analysis in Alberta and Nova Scotia. They found that tobacco producers were complying with “the letter of the law,” in that no products were packaged as menthols, but post-ban menthol replacements kept the same color and design for easy identification as a similar product but no longer containing menthol.

Stokolsa [19] evaluated the possibility of a black market for menthol and non-menthol cigarettes in Nova Scotia following the 2015 menthol ban by applying a time series analysis to Provincial Tax Commission 2012–2018 data on the number of illicit cigarettes seized. They did not find evidence of increased seizures of menthol or non-menthol cigarettes, suggesting that a black market for menthol cigarettes did not develop.

### Hypothetical menthol ban

Twelve studies considered hypothetical menthol bans, which explored the behavioral intentions of individuals in response to a proposed ban. Nine studies used traditional survey methods: six predominantly surveyed adults (generally ages 18 and above) and three exclusively surveyed young adults (generally up to age 34). Three studies used non-survey methods: one employed focus groups and two conducted discrete choice experiments, to assess individual intentions in response to a ban (Table 2).

**Table 2:** Studies that report results related to hypothetical menthol bans

Author, Year	Location	Age group	Study Design (Theme)	Sample Size	Ban Specifics (Implementation Date)	Data Sources	Main Outcomes	Results
Buckell, J. 2019 [23]	US	18–64	Cross-Sectional (Individual Intentions)	*N* = 2031 smokers and recently quit smokers	Hypothetical national ban on menthol cigarettes and menthol/flavored e-cigarettes	Discrete choice experiment considering various combinations of menthol cigarette and menthol or fruit/sweet e-cigarette bans.	Change in product choice shares	Current and former smokers preferred cigarettes to e-cigarettes, but differences by age, race, and education exist. Banning menthol cigarettes would produce the greatest reduction in the choice of cigarettes (−5.2%), but with an accompanying increase in e-cigarette use (3.8%). Banning flavors, including menthol, in e-cigarettes without banning menthol cigarettes would result in an 8.3% increase in the use of combustible cigarettes and an 11.1% decline in e-cigarette use. Banning all flavors across all products would increase ‘opting-out’ the most (5.2%), but would also increase cigarette choice by 2.7%.
D’Silva, J. 2015 [24]	Minnesota, US	18+	Cross-Sectional (Individual Intentions)	*N* = 1158 current menthol smokers (100+ cigarettes and smoked some or everyday with menthols as their usual brand)	Hypothetical ban on menthol cigarettes	Minnesota Adult Tobacco Survey	Cessation and product switching	Nearly half (46.4%, [37.9, 54.9]) of menthol smokers responded that they would quit smoking. Approximately one-fourth of menthol smokers (26.6%, [19.0, 34.1]) reported that they would switch to non-menthol cigarettes; 12.3% [6.3, 18.3] would switch to menthol e-cigarettes, 5.8% [1.8, 9.7] would buy menthol cigarettes online, 2.8% [0.4, 5.2] would switch to some other menthol tobacco product, 2.7% [0.0, 6.0] would buy menthol cigarettes from another country, and 1.5% [0.0, 3.8] would switch to some other non-menthol tobacco product. African-American menthol smokers were more than twice as likely to report an intention to quit in the event of a ban (76.0%, [57.6, 94.3]) compared to their white counterparts (30.3%, [21.7, 38.9]) (RR = 2.5, [1.7, 3.6], *p* < .001).
Guillory, J. 2019 [25]	US	18+	Cross-Sectional (Individual Intentions)	*N* = 1197 menthol smokers	Hypothetical national ban on menthol cigarettes	*RTI iShoppe* virtual convenience store. Four types of virtual ban: a) no ban; b) replacement of menthol cigarettes and ads with green versions; c) menthol cigarette ban; d) all menthol tobacco products ban.	Consumer behavior in response to bans, brand loyalty, and perceived response.	Cigarette purchases were higher in the no ban (59%) and green conditions (59%) than the menthol cigarette ban (49%) and all menthol ban conditions (47%). Menthol cigarette purchases were highest in the no ban condition (50%). Other tobacco product (OTP) purchases were low across conditions, ranging from 16 to 17%, with 2–3% of all tobacco purchases being menthol e-cigarettes. Purchases of a substitute cigarette brand were highest in the menthol cigarette ban condition (61%) and the all menthol ban (60%).OTP purchases were similar across four scenarios, suggesting menthol bans may not increase OTP purchases.
Hartman, A. 2011 [22]	US	18+	Cross-Sectional (Individual Intentions)	*N =* 10,441 (*n*= 2887 regular menthol smokers)	Hypothetical national ban on menthol cigarettes	Tobacco Use Supplement-current Population Survey 2010	Cessation and product switching	39% of usual menthol users (30% of the smokers’ sample) reported they would quit and not switch to an alternative tobacco product. This included 40.6% of the 18–44 year-olds and 36.7% of the 45+. Women and non-Hispanic blacks report less intention to quit compared to men and non-Hispanic whites.
O’Connor, R. 2012 [26]	US	14–65	Cross-Sectional (Individual Intentions)	*N* = 417 (*n* = 170 menthol users).	Hypothetical national ban on menthol cigarettes	Global Market Institute, Inc. online survey panel, July 2010. Sample stratified smokers and non-smokers by age group	Cessation and product switching	More than 35% of menthol smokers reported the intention to quit smoking, 25% plan to seek out menthol cigarettes. Demand elasticity for non-menthol products in menthol smokers was 50% higher than for non-menthol smokers.
Pacek, L. 2019 [27]	US	18–29	Cross-Sectional (Individual Intentions)	*N* = 240 (*n* = 126 menthol users)	Hypothetical national ban on menthol cigarettes (also considered low nicotine content cigarettes)	Amazon Mechanical Turk survey, 2017. Dual (combusted and e-cigarette) users.	Use of e-cigarettes in response to menthol cigarette ban	Approximately 25% (aged 18–29) would plan to quit and 32.5% would reduce the amount smoked. Approximately 30% of menthol cigarette/e-cigarette dual users reported an intention to increase e-cigarette use following a menthol ban.
Pearson, J. 2012 [28]	US	18+	Cross-Sectional (Individual Intentions)	*N* = 2649	Hypothetical national ban on menthol cigarettes	Data from the Knowledge Panel. Never, former and current smokers. Stratified by sex, ethnicity, age group, education, health status, intention to quit and quit attempts	Attitudes towards menthol bans; cessation and product switching.	Menthol smokers were more likely than non-menthol smokers to disagree with a menthol ban (50.5% vs. 31.2%; *P* < 0.001). 38.9% of menthol smokers said that they would quit, 13% would switch to a nonmenthol cigarette, 25% would switch to regular cigarettes and try to quit.
Rose, S. 2019 [29]	US	18–34	Cohort (Individual Intentions)	*N* = 806	Hypothetical national ban on menthol cigarettes	Truth Initiative Young Adult Cohort from 2011 to 2016	Cessation and product switching	Switching to non-menthol cigarettes was most common post-ban intention (mean of 32.3% across multiple waves). 30.8% did not know what they would do in response to a menthol ban. 23.5% reported they would quit and 10.7% reported the intention to use an alternative tobacco product.
Wackowski, O. 2014 [30]	US	18–34	Cross-Sectional (Individual Intentions)	*N* = 2871 (*n* = 619 menthol users)	Hypothetical national ban on menthol cigarettes	National Young Adult Health Survey, 2011. Menthol smokers	Cessation and product switching	64% would try to quit smoking, 18% would switch to non-menthol cigarettes, 15.7% would switch to OTP, and 1% didn’t know.
Wackowski, O. 2015 [31]	US	18+	Cross-Sectional (Individual Intentions)	*N* = 519 (*n* = 187 menthol users)	Hypothetical national ban on menthol cigarettes	Online survey panel, April 2014. Stratified by ethnicity.	Cessation and product switching	28.4% would try to quit smoking; 45.9% switch to nonmenthol cigarettes; 3.9% would switch to OTP; 15.1% would switch to menthol e-cigarettes.
Wackowski, O. 2018 [32]	New Jersey, US	18–24	Cross-Sectional (Individual Intentions)	*N* = 45 (in 6 focus groups)	Hypothetical ban on menthol cigarettes	Focus groups from Dec 2014 to Mar 2015	Attitudes and perspective towards menthol cigarettes	59.1% indicated that either all (34.1%) or most (25%) of their first few cigarettes were mentholated.Easy accesses to loosies influenced menthol use (particularly among African Americans). Several people noted that they were willing to smoke a friend’s non-menthol cigarette if they didn’t have their cigarettes. Many participants were highly skeptical that a ban could be effective, believing that people would still find a way to get menthol cigarettes, either on the “black market” or by making bootleg versions.Some stated that a ban would not make much of an impact on them because they would just switch to non-menthol cigarettes. However, others thought a ban might motivate them to quit and increase their likelihood of doing so.
Zatoński, M. 2018 [33]	Europe	18+	Cross-Sectional (Individual Intentions)	*N* = 10,760 smokers (100+ cigarettes in their lifetime)	Hypothetical ban on menthol cigarettes	Smokers from 8 European countries from the International Tobacco Control Policy Evaluation	Cessation and product switching	When asked about their intended behavior following a hypothetical ban, most respondents reported intending to find menthol cigarettes regardless of the ban (27.3%; 95% CI 23.7–31.3), 20% reported an intention to switch to another product (95% CI 16.9–23.4), 17.6% reported an intention to reduce their smoking amount (95% CI 14.5–21.1), 16.0% reported an intention to quit (95% CI 13.3–19.2), and the remainder reported that they would ‘do something else’ or did not know.

Using the 2010–2011 Tobacco Use Supplement-Current Population Survey, Hartman et al. [22] reported that, among US adult menthol smoker, 39% said that they would attempt to quit, 36% would switch to non-menthol cigarettes and 8% would switch to an alternative tobacco product in response to a menthol ban. African Americans, women, and younger individuals (aged 18–44) were more likely to report intentions to quit (47, 42, and 41% respectively) than white, male, or older individuals (34, 36, and 37% respectively).

Four studies employed online surveys of predominantly adult smokers. D’Silva et al. [24] considered information from 1158 current menthol smokers completing the Minnesota Adult Tobacco Survey. They reported that nearly half of menthol smokers (47%) would quit smoking, 27% would switch to non-menthol cigarettes, 12% would switch to menthol e-cigarettes, 8.5% would buy menthol cigarettes online or from another country, and 4% would switch to another tobacco product with or without menthol. Additionally, they found that African American menthol smokers were twice as likely to report an intention to quit when compared to white menthol smokers (76.0% vs. 30.3%, respectively). Using a convenience sample of 471 adolescent and adult menthol smokers aged 14–65, O’Connor et al. [26] found that 17% would not consider using non-menthol cigarettes, 35% would attempt to quit, and 25% would seek out menthol cigarettes. Some would consider using alternative menthol products, such as cigars (12%) or smokeless tobacco (18%). Pearson et al. [28] surveyed 6792 current smokers and 2649 former smokers from the US nationally representative KnowledgePanel cohort. They found that, among menthol smokers, 39% would attempt to quit smoking following a menthol ban, 25% would switch to non-menthol cigarettes and try to quit, while only 13% of menthol smokers would switch to non-menthol cigarettes without attempting to quit. Of 519 current adult cigarette smokers including 36.3% were menthol smokers, Wackowski et al. [31] found that 46% of menthol smokers would switch to non-menthol cigarettes, 28% intended to quit smoking, 15% would switch to menthol e-cigarettes, and 4% would switch to other tobacco products.

Zatoński et al. [33] conducted a cross-sectional survey of 10,760 adult current smokers in 8 European countries. The proportion of smokers who reported menthol as their usual brand was 7.4% combined across the 8 countries, with a range from 12% in England to 0.4% in Spain. The proportion was higher among women than men in all eight countries. When asked about their intended behavior following a hypothetical ban, 27% of respondents reported an intention to find menthol cigarettes regardless of the ban, 20% reported an intention to switch to another product, 18% reported an intention to reduce the amount smoked, 16% reported an intention to quit, and the remainder reported that they would ‘do something else’ or did not know. The intention to switch to another product ranged from 45% in Romania to 17% in Greece, the intention to find menthol cigarettes ranged from 35% in the Netherlands to 12% in Poland, and the intention to quit smoking ranged from 17% in England to 2% in Germany.

Among cross-sectional studies focused on US young adult menthol smokers, Pacek et al. [27] found that approximately 25% (aged 18–29) would plan to quit and 32.5% would reduce the amount smoked, while Wackowski et al. [30] reported that 64% (aged 18–34) would plan to quit, 18% would switch to non-menthol cigarettes, and 16% would switch to an alternative tobacco product. Pacek et al. [27] also surveyed participants regarding e-cigarette use, finding that approximately 30% of menthol cigarette dual users reported an intention to increase e-cigarette use following a menthol ban. Rose et al. [29] examined responses among the Truth Initiative Young Adult Cohort from 2011 to 2016 (aged 18–34). They found that 32.3% of respondents would switch to non-menthol cigarettes, 23.5% would consider quitting, and 10.7% would switch to another tobacco product. No statistically significant changes were seen in the responses between 2011 and 2016, except for those who reported the intention to switch to an alternative tobacco product which saw an increase from 7.4 to 13.2%.

Three studies used designs other than surveys to assess individual behavior in response to a hypothetical ban. Wackowski et al. [32] conducted focus groups among 45 young adults in New Jersey aged 18–24, from December 2014 to March 2015. The groups were asked about menthol preferences, smoking behavior, and thoughts on a potential ban. The majority (59.1%) initiated smoking with exclusive or predominant use of menthol cigarettes. Respondents disapproved of a menthol ban claiming that smoking is an individual’s choice, but indicated that it would help them to quit smoking [32].

Guillory et al. [25] employed a virtual store based discrete choice experiment to examine 1197 menthol smokers’ purchase behavior in response to four scenarios: 1) no ban; 2) replacement of menthol cigarettes with green versions (green packages of non-menthol cigarettes, similar to those seen following Canada’s menthol ban); 3) menthol cigarette ban; and 4) all menthol tobacco product ban. They found that cigarette purchases were higher in the no ban (59%) and green conditions (59%) than the menthol cigarette ban (49%) and all menthol ban (47%) scenarios. Purchases of cigarettes that were not the participant’s usual brand were highest in the menthol cigarette ban (61%) and the all menthol ban (60%) scenarios. Other tobacco product purchases were low (16–17%) across all conditions.

Buckell et al. [23] conducted a discrete choice experiment with an online sample of 2031 adult smokers and recent quitters aged 18–64 to estimate the impact of flavor bans, including menthol, on preferences and the demand for cigarettes and e-cigarettes. The results of the discrete choice experiment suggested that if all flavors including menthol were banned from cigarettes and e-cigarettes, the proportion of individuals choosing combustible cigarettes or no products would increase relative to the current national US policy. They found a 6% increase in combustible cigarette selection, a 30% increase in no products selected, and a 21% decrease in e-cigarettes selection. The menthol cigarette ban alternative where flavored e-cigarettes (including menthol) were still allowed resulted in the largest reduction in cigarette use (12% reduction), with a concurrent increase in e-cigarette use (10%) and no products selected (9%).

### Implemented flavor ban

Six studies examined the effects of implemented flavor bans, none of which included menthol. Two studies considered individual behavior, three explored changes in sales, and one explored compliance (Table 3).

**Table 3:** Studies that report results related to implemented flavor bans

Author, Year	Location	Age group	Study Design (Theme)	Sample Size	Ban Specifics (Implementation Date)	Methods	Main Outcomes	Results
Chaiton, M. 2019 [34]	Canada	NA	Time Series (Sales)	NA	2009 National ban on flavored cigarettes and little cigars, except menthol	Quarterly sales data from Health Canada from 2004 to 2015.	Sales of cigars	Post-ban, a there was a decline in flavored cigars sales after 2009 of 59 million units. Incomplete substitution with an increase of 9.6 million in non-flavored cigars.
Courte-manche, C. 2017 [35]	US	11–19	Trend Analysis (Individual Behavior)	Not Specified	2009 National ban on flavored cigarettes, except menthol	National Youth Tobacco Surveys from 1993 to 2013. Controlled by age, sex, and race, tax-inclusive price indices for cigarettes and Other tobacco products (OTP).	Type of tobacco product use in the past 30 days. Cigarettes, menthol cigarettes, or OTP (cigars, smokeless, pipes) or non-cigarette tobacco products	Post-ban, there was a decrease of 6% in the probability use of any tobacco products. Adolescents were more likely to choose menthol cigarettes, cigars and pipes. Substitution to other tobacco products increased by 14%.
Delnevo, C. 2015 [13]	US	NA	Trend Analysis (Sales)	NA	2009 National ban on flavored cigarettes, except menthol	Nielsen’s convenience store data on clove cigars, 2009–2012.USDA/ GATS data on imported cigars and cigarettes from 2008 to 2012. Quantity and value of cigars and cigarettes from Indonesia 2008–2012	Sales and total imports of clove cigars. Marketing Strategies.	Kretek International’s development of clove cigar started in 2007 by changing only the product’s wrapper from cigarette to cigar. Kretek took advantage of the disparities between cigarette and cigars warning labels and excise tax. Clove cigars sales increased from 444,000 in 2009 to 6.7 million in 2012 (1400%). Cigars imports increase to > 626 million sticks by 2012.
Delnevo, C. 2017 [36]	US	NA	Trend Analysis (Sales)	NA	2009 National ban on flavored cigarettes, except menthol	US Nielsen convenience store sales data from 2008 to 2015 to identify cigar’s specific brand, flavor, and packaging characteristics	Sales of cigars packaging characteristics, or flavors.	From 2008 to 2015, unit sales of cigars steadily increased from 994.2 million to over 1.5 billion. More than half of cigars sold in 2015 were flavored, an 8.5% absolute change in market share. From 2008, the number of unique flavor names doubled during this period, from 108 individual flavors to 250 by 2015. Sales of single and 5-pack cigars fell in favor of 2–3 packs which rose from 1% in 2008 to 40% in 2015.
Jo, C. 2014 [37]	US	NA	Pre and Post Analysis (Compliance)	*N* = 200 internet cigarette vendors	2009 National ban on flavored cigarettes, except menthol	Internet tobacco vendors product availability, Internet Cigarette Vendor study 2009, 2010, and 2011	Sales of flavored tobacco products	Post-ban, 89% of vendors continued to sell flavored products however, the majority (67.8–82.5%) of these retailers were international. Percentage of flavored US vendors fell from 50.9% in 2019 to 28.6%. Vendors were 1.71 times more likely to sell flavored little cigars in 2010 compared to 2009; and 5.50 times more likely to sell clove cigarettes. The percentage of vendors selling clove cigarettes and cigars increased from 20.6% in 2009 to 25.5% in 2010 and then decrease to 15.5% in 2011.
Nguyen, H. 2014 [38]	Canada	15–65	Trend Analysis (Individual Behavior)	*N* = 46,000 observations	2010 Ban on flavored cigarillos and unflavored packs with > = 20 units	2007–2011 Canadian Tobacco Use Monitoring Survey	Change in young person’s use of cigarillos and regular cigars	For entire sample, 39% reported ever smoking cigarillos and 9% reported past 30-day use. Past 30-day use of cigarillos by those aged 15–24 declined from 13.7 to 9.3% (*p* = 0.000) for male respondents and from 5.3 to 3.3% (*p* = 0.001) for female respondents. Reductions in cigarillo use for the older age group were not statistically significant. Regression analysis found a 2.3 percentage point decline in past 30-day cigarillo use among young people (22% relative decline); a 4.3 percentage point increase in past 30-day abstinence. For youth, all cigar ever use declined by 2.2 percentage points (5.1% relative reduction) and by 3.1% for ever use of cigarillos (8% relative decline).

#### Individual behavior

Two studies considered the impact of an implemented tobacco flavor ban on current tobacco use and initiation. Courtemanche et al. [35] analyzed data from the National Youth Tobacco Survey 1999–2013 and found that in the 4 years following the FDA’s 2009 non-menthol flavored cigarette ban, there was a 17% decrease in the past 30-day use of cigarettes in the overall youth population. A 14% increase in cigar, smokeless tobacco, and pipe use indicated substantial substitution into related products. Overall, they found a 6% decrease in the probability of all youths using any tobacco product in the 4 years post-ban. Nguyen et al. [38] evaluated the effects of banning flavored cigarillos in Canada in 2010. Using Canadian Tobacco Use Monitoring Survey data from 2007 to 2011, they found that past 30-day use of cigarillos among those aged 15–24 declined in relative terms by 32% among men and 38% among women, and regression analyses found that there was a 22% decline in past 30-day cigarillo use. Changes for older age groups were lower and did not reach statistical significance. Substitution for other products was limited, with regression analyses indicating no evidence of higher cigarette smoking. Overall, among respondents aged 15–24, the policy was found to significantly reduce all cigar type ever use 5.1% in relative terms, and ever use of cigarillos by 8%.

#### Sales

Chaiton et al. [34] evaluated the 2009 Canadian ban on all non-menthol flavor additives in cigarettes and cigars under 1.4 g. They applied an interrupted time series analysis on quarterly wholesale data from Health Canada for 2004 to 2015 on the sale of flavored cigars. The study observed a 59 million-unit decline in flavored cigars from a pre-intervention level of 150 million units (~ 39% reduction). At the same time, there was an increase in the sales of non-flavored cigars by 9.6 million units from the initial level of 35 million units (~ 27% increase). Overall, we calculate that the ban resulted in a net reduction in all cigar sales of approximately 27% ((59–9.6)/(150 + 35)*100%).

Two studies examined changes in flavored cigar sales following the 2009 US flavored cigarette ban (excluding menthol). Delnevo et al. [13] used 2008 to 2012 Nielsen convenience store and US Department of Agriculture data to track changes in clove cigar sales, which were marketed as a post-ban substitute for clove cigarettes. They found a 1400% sales increase in clove cigars in 2012 compared to 2008. In a separate study, Delnevo et al. [36] applied a trend analysis of Nielsen convenience store data from 2008 to 2015 to examine the post-ban change in cigar sales. Over this period, they found an increase in sales of all cigars from 994 million units in 2008 to 1.5 billion units in 2015. Additionally, they reported that more than half of cigars sold in 2015 were flavored, representing a 46.5% increase in flavored cigar sales from 2008 levels.

#### Compliance

One study evaluated compliance with the FDA’s flavored cigarette ban. Jo et al. [37], conducted a pre- and post-analysis of the availability of flavored cigarettes following the FDA’s 2009 flavor ban using a sample of 200 internet cigarette vendors from 2009 to 2011. They found that 89% of internet vendors continued to sell banned products or products with misleading names and descriptions (i.e. “light,” “mild,” “low,” and similar terms). The majority of the vendors analyzed were not US-based, but 29% of US vendors continued to sell flavored cigarettes following the ban compared to 51% pre-ban, while 96% of international vendors continued to sell products banned in the US. Additionally, compared to 2009, internet vendors were more likely to sell flavored little cigars.

Table 4 contains a summary of the range of results regarding sales, initiation, cessation, and product switching for implemented and hypothetical bans, as well as for (non-menthol) flavor bans.

**Table 4:** Summary results of implemented and hypothetical menthol and flavor bans on sales and individual behavior

	Implemented Menthol Ban (Actual Effects)	Hypothetical Menthol Ban (Intended Effects)	Implemented Flavor Ban – Cigarettes Only	Implemented Flavor Ban – All Tobacco Products
Sales change (banned product)	~ 100% reduction	NA	NA	39% reduction in flavored cigar sales
Sales change (all tobacco products)	11% reduction	NA	47% increase in cigar sales; 1400% increase in clove cigar sales	27% reduction in all cigar sales
Quit Attempt	29–63%	24–64%	NA	NA
Successful Quit	24%	NA	NA	NA
Switch to other tobacco product	28.2–76.1%	11–46%	14%	0–11%
Switch and attempt to quit	NA	20–25%	NA	NA
Switch to e-cigarettes	29.1%	12–30%	NA	NA
Find product regardless of ban	NA	9–25%	NA	NA
Reduced Odds of Trying Any Tobacco Product	NA	NA	6%	NA
Reduced Odds of Trying Cigars	NA	NA	NA	5%

## Discussion

We aimed to assess the potential changes in individual and industry behaviors resulting from a US menthol cigarette ban. We found 18 studies that directly considered an actual or hypothetical cigarette menthol ban and six studies that examined a non-menthol flavor ban. The studies find major impacts or potential impacts of a ban on smoking behaviors.

Regarding smoking cessation, an Ontario menthol cigarette ban study provided the most direct results of an actual ban, with 24% of daily menthol smokers quit by one-year post-ban [17]. Another Ontario study found that all cigarette sales declined by 11%, with minimal substitution of non-menthol for menthol cigarettes [16]. In addition, studies of non-menthol flavor bans found that sales of flavored cigars fell by 39% with minor substitution to non-flavored products [34]. While US studies have been limited to evaluations of intentions in response to a proposed menthol ban, they indicate that 25–46% of adult menthol smokers would quit [22, 24, 26, 28, 31], with up to 65% intending to quit among young adults [27, 29, 30]. Based on our review, a credible range of 11 to 45% of current U.S. menthol smokers would quit smoking in response to a menthol cigarette ban [17, 18], possibly higher among young adults. These results are consistent with previous reviews of observed cessation rates among menthol compared to non-menthol smokers in the absence of a ban [8, 9, 39] and estimates derived for the simulation of a menthol ban [40].

Studies also indicate that the impact of a menthol ban on current menthol smokers is likely to depend on the availability of e-cigarettes. Three of the hypothetical ban studies found that 15 to 30% of menthol smokers intended to substitute e-cigarettes for menthol cigarettes [24, 27, 31], and a more recent discrete choice study [23] also found that e-cigarette substitution could play a major role. These results are consistent with studies that indicate e-cigarettes may provide a substitute for smokers as a cessation aid [23, 41, 42] and may substitute for the initiation of cigarette smoking [43]. The ability of e-cigarettes to substitute for cigarettes is also consistent with recent studies of e-cigarette demand, where e-cigarette use has been found to be relatively responsive to e-cigarette [44–47] and cigarette prices [44, 46, 48], thus indicating their substitutability for cigarettes. The studies we identified were generally conducted at the early stages of e-cigarette use. With improvements in e-cigarette technology, greater substitution of e-cigarettes for menthol cigarettes may be expected. However, the ability to substitute e-cigarettes for cigarettes is likely to be reduced if e-cigarette flavors, especially menthol and mint, are banned. While the effects of a menthol cigarette ban on cigarette smoking rates may be intensified by the ability to switch to e-cigarettes, any associated increase in e-cigarette use should consider the potential health implications associated with their use [49]. In addition, Altria began marketing their heat-not-burn product IQOS including menthol and mint flavors [3, 50], which is also likely to affect the impact of menthol bans on smoking rates.

A menthol ban may also impact non-menthol smokers and those who occasionally but do not primarily use menthol cigarettes, as suggested by changes in cigarettes sales in Canada [16]. For example, through social network effects resulting from reduced menthol cigarette use by their peers, youth and young adults who may have initiated smoking non-menthol cigarettes may be less likely to initiate smoking, and friends or family members smoking non-menthol cigarettes may be more likely to quit if friends or family member smoking menthol cigarettes quit. Non-menthol smokers may also respond to the publicity surrounding a menthol ban. In particular, it is unknown whether those smokers who only occasionally smoke menthol cigarettes and are not generally considered menthol smokers may be more motivated by the ban to stop smoking. Further study is warranted to examine the potential impact of a menthol ban on non-menthol and non-regular menthol smokers.

None of the menthol ban studies directly estimate the effect of a ban on smoking initiation. However, a study of flavor bans indicates that youth cigarette tobacco initiation was reduced by 6% [35]. Additionally, another three studies (29–31) of young adults found larger percentages of menthol smokers quitting or switching to e-cigarettes than studies of all adults, suggestive of less cigarette smoking by those at ages when initiation patterns are still being formed. Impacts on initiation are also consistent with recent reviews of actual menthol use, which have found that youth are particularly likely to begin with menthol cigarettes and those who initiate smoking with menthol are more likely to progress to established smokers than those who initiate with non-menthol tobacco products [8, 51, 52]. Further, a recent study found that initiation with flavored tobacco products (including menthol) was associated with a 32% higher prevalence of established tobacco use among adult users [53]. Nevertheless, further study is warranted on the effects of a menthol ban on smoking initiation.

The impact of a menthol ban will also depend on compliance with the ban by individuals, sellers, and manufacturers. Compliance with the menthol cigarette ban in Canada was high, with all packaging complying with the letter of the law [14, 15] and seizures of illegal menthol cigarettes limited [19]. However, studies on compliance with flavor bans in the US showed some loopholes. A study of internet vendors following the FDA’s flavor ban found that 89% of all vendors and 28% of US vendors continued to sell banned products [37], while manufacturers will attempt to develop replacement products which could reduce the effectiveness of a ban, as seen with the increased sales of clove cigars [13]. Despite potential noncompliance through internet sales, compliance may be higher with greater enforcement efforts.

We did not consider bans with exclusions for some locations, due to loopholes regarding the ability to purchase at other locations and their more limited relevance to the likely components of national menthol cigarette ban. We identified nine such studies, all of which examined local regulations that limited the sale of flavored tobacco products with exceptions for retail establishments selling to those over 21 years of age or tobacco shops. These are summarized in Additional file 1: Table 1. Of the nine excluded studies, one study found that adolescents had a 37% lower odds of ever trying flavored tobacco products and a 28% lower odds of ever using tobacco products post ban [54], two studies found that sales of other tobacco products fell [55, 56], and six considered compliance [57–62]. The studies of compliance indicated mixed levels of compliance, with flavor products still available in up to 50% of retailers that were not allowed to sell flavored tobacco and smaller stores often continuing to carry banned products with staff oblivious to the ban [58, 60–62]. However, compliance was found to increase over time following the ban [60] and was higher in areas where investments are made in the education of store owners and staff and when there was increased enforcement [59].

We also did not consider results regarding support for bans. Nonetheless, studies indicate that the general populace and, more importantly, menthol smokers were not strongly opposed to a ban [28, 63, 64]. Some studies have even found menthol smokers to be supportive of a ban in the hopes that it would help further motivate them to quit [64], reducing concerns about compliance at the individual level.

While the evidence indicates strong potential for a menthol ban to impact smoking behaviors, our review is subject to limitations. First, not all evidence was US-based, so the summarized results may not be directly relevant to the US, as highlighted by the high levels of intercountry variation in menthol use and reactions to a ban found by Zatoński et al. [33]. The US studies of smoking behaviors were based on surveys of intended reactions to a hypothetical ban and may overstate actual behaviors. In addition, many of these studies depend on particular services (e.g., Knowledge Networks) or other online convenience samples, which rely on similar strategies for selecting the participants in their surveys. These studies may not be generalizable to the US population as a whole.

There are also limitations related to the outcomes reported in the original studies. For example, the measure of cessation reported by Chaiton et al. [17] in their study of Canada’s menthol ban was a statement of “not at all” in response to being asked about their current use of menthol or non-menthol cigarettes. No results to follow-up questions about how long the individual had quit smoking or biochemical verification of cessation were reported, thereby potentially overestimating the effect of a ban. An additional limitation is that subgroup data for many of the populations with the highest prevalence of menthol use was not presented (notably, African Americans and women), potentially masking differential effects.

Finally, our review is limited in its ability to report results corresponding to the ongoing shifts in the tobacco market and regulatory environment. In particular, all of the studies cited were conducted prior to the more recent increase in the adoption of e-cigarettes and the marketing of IQOS. While these products may enhance the effect of a menthol ban by providing a menthol alternative, regulations that reduce their availability may reduce the impact of a menthol ban. In addition, cigarette smokers may substitute flavored smokeless tobacco, dissolvables or cigars, especially little cigars, if flavors are not restricted for those products [65]. In general, the impacts of a menthol ban will depend on flavor restrictions as they are applied to all nicotine delivery products, and, more generally, on the impact of regulations on future technological changes and the marketing of those products.

## Conclusions

In conclusion, based on our literature review, we estimated substantial impacts of a menthol ban on smoking cessation and initiation. The impacts are expected to be greater if compliance with the ban is high and if e-cigarettes, especially those that are menthol and mint-flavored, are available. While a substantial literature has considered the impact of menthol use on initiation and cessation, further studies should consider the effects of menthol cigarette bans that have already been implemented in local areas of the US, paying particular attention to smoking initiation, the role of e-cigarettes and compliance. Nevertheless, the evidence to date indicates that a menthol cigarette ban, especially if implemented nationally, provides an important opportunity to improve public health by reducing smoking-attributable diseases.

## Supplementary information

**Additional file 1: Table 1.** Studies of flavored other tobacco product bans that excluded over 21 establishments.

## Data Availability

Data sharing is not applicable to this article as no datasets were generated or analyzed during the current study.

## Abbreviations

FDA: Food and Drug Administration
US: United States of America
