# Supplementary material for: The actual and anticipated effects of a menthol cigarette ban: a scoping review
Source: BMC Public Health. 2020 Jul 9;20:1055. doi: 10.1186/s12889-020-09055-z (PMC7346606; doi:10.1186/s12889-020-09055-z)
Supplement: Supplementary file 1 — Additional file 1: Table 1. Studies of flavored other tobacco product bans that excluded over 21 establishments. [file 12889_2020_9055_MOESM1_ESM.docx]

**Appendix Table 1:** Studies of flavored other tobacco product bans that excluded over 21 establishments.

| **Author, Year** | **Location** | **Age group** | **Study Design**  **(Theme)** | **Sample Size** | **Ban Specifics (Implementation Date)** | **Methods** | **Main Outcomes** | **Results** |
| --- | --- | --- | --- | --- | --- | --- | --- | --- |
| **Behavior and Sales** | | | | | | | | |
| Rogers, T. 2017 (8) | New York City, US | NA | Quasi-experimental  **(Sales)** | NA | 2009 City-wide ban on all flavored tobacco products excluding menthol to adult-only tobacco shops (enforced 2010) | NYC (ban), NYC proximal comparison area (PCA) and the US (no ban) Nielsen convenience store sales data from 2010 to 2014 | Sales of total units of flavored cigars, smokeless, roll your own and total cigars. | Post ban, NYC cigar sales fell -22.3%, smokeless -997.6%, roll your own -42.5% and total cigars by -7.4%. PCA and US flavored cigar sales increased by 3.2% and 14.6%; all flavored products increase by 2.2% and 10.9%, and total cigars by 9.8% and 12.0% respectively. |
| Rogers, T. 2019 (9) | Province, Rhode Island. US | NA | Time Series Analysis  **(Sales)** | NA | 2012 City-wide ban on all flavored tobacco products excluding menthol except in certain adult only facilities (enforced 2013) | Providence (ban) and rest of Rhode Island comparison area (ROS) (no ban) Nielsen retail data from 2012 to 2016. | Unit sales | Post ban, Providence, immediately after there was a-48% reduction in all flavored non-cigarette products cigarette tobacco products Average weekly sales of all-non-cigarette tobacco products flavored decreases 18%. Changes were mainly driven by a reduction in cigar sales (-31%). There was a drop from 1.0% to 0.5% in the share of menthol/mint flavors cigars. ROS, immediately there was an 8% increase in all flavored non-cigarette products. No change in average weekly sales of all-non-cigarette tobacco products.  Pre and post-ban changes in average weekly unit sales of tobacco-flavored cigars in Providence and ROS control area (+11.3% and −19.5%, respectively). |
| Farley, S. 2017 (2) | New York City, US | 13 to 17 years | Time Series and Trend Analysis  **(Sales & Individual Behavior)** | N=1000 unique products and N=922 stores  N=1708 in 2010 and N=8814 in 2013. | 2009 City-wide ban on all flavored tobacco products excluding menthol to adult only tobacco shops  (enforced 2010) | Retail Tobacco Sales data from five New York City (NYC) boroughs from 2008 to 2012; and NYC Youth Risk Behavior Survey (YRBS) 2010 (pre-ban) and 2013 (post-ban). | Change in flavored and non-flavored product use overall and by product type. | Post ban, sales of flavored tobacco declined 87%, driven by cigars sales. The sales of non-flavored tobacco products showed a non-significant increase, however, statistically, significant increases were found in sales of cigars and roll your own.  Post-ban, adolescents had 37% lower odds of ever trying flavored tobacco products and 28% lower odds of ever using tobacco products (OR 0.72). |
| **Compliance** | | | | | | | | |
| Brock, B. 2018 (1) | Minneapolis and Saint Paul, MN | NA | Pre and Post Analysis **(Compliance)** | 92 grocery and convenience stores | Restricted the sale of flavored tobacco to adult-only tobacco shops (2016) | Observational assessments at stores in Minneapolis (n=41), Saint Paul (n=37) and a comparison city of Brooklyn Park (n=14) between 2015 and 2017. | Compliance with flavor restriction, change in the proportion of inventory that is menthol, other flavors, or unflavored; exterior advertising, and availability of tobacco with ambiguous flavor names. | Following implementation, fewer stores sold flavored tobacco products in Minneapolis (85.4% vs 39.0%) and Saint Paul (97.3% vs 8.1%). Brooklyn Park convenience/grocery stores sold flavored tobacco than the stores in both Saint Paul and Minneapolis after policy implementation. Saint Paul saw no significant change in the presence of tobacco with ambiguous flavor names (80.5% vs 61.5%) while Minneapolis saw a reduction (80.5% vs 61.5%). There was no statistically significant difference between the post-intervention presence of ambiguous flavor named products in Saint Paul or Minneapolis when compared to the control city, Brooklyn Park (57.1%). The presence of exterior advertising remained relatively constant. |
| Farley, S. 2018 (3) | New York City, US | NA | Cross-Sectional  **(Compliance)** | N=19 tobacco product (n= 16 with flavor names. n= 3 without flavor names) | 2009 City-wide ban on all flavored tobacco products excluding menthol to adult only tobacco bars  (enforced 2010) | NYC purchase of non-cigarette tobacco products. Feb/Mar 2015. Each product content analyzed in triplicate | Content of flavor chemicals in non-flavored tobacco products. | Tobacco products without flavor names are flavored. Fourteen tobacco products were found to contain flavor chemical levels higher than the lowest total level (0.313 mg/g) found in 18 tobacco products with flavor names previously analyzed |
| Kephart, L. 2019(4) | Boston, MA | NA | Pre and Post Analysis **(Compliance)** | 488 retailers at baseline 469 at follow-up | Restricted the sale of flavored tobacco to adult-only tobacco shops (2016) | Observational assessment at stores in Boston | Compliance with flavor restriction, types of flavor products sold, and the presence of flavor tobacco advertising. | At baseline 88.6% of retailers sold flavored tobacco products; retailers sold an average of 19.5 types of flavored products, and advertising for flavored tobacco products was present at over half (58.9%) of tobacco retailers at baseline. Post-ban 14.4% of youth-accessible tobacco retailers still sold flavored tobacco products compared with 100% among all complete case retailers at baseline; the average number of flavored products being sold at follow-up was 0.39 products among all stores, and advertising decreased by 28.6 percentage points between the baseline and follow-up period. Among stores who were still selling flavored tobacco products at follow-up (n=51), the average number of flavored products being sold was three products. At the stores not in compliance, 72.5% did not know a product was in violation of the policy. |
| Kingsley, M. 2019 (5) | Massachusetts | NA | Pre and Post Analysis **(Compliance)** | 38 communities that implemented restrictions, (18 in wave 1 and 20 in wave 2). 234 control communities | Numerous local restrictions on the sale of flavored tobacco (excluding mint and menthol) to adult-only locations. Implemented in Massachusetts from July 2015 to March 2017. | Massachusetts Tobacco Control Program pricing survey; two sets of pre and post-implementation analyses compared to localities without restrictions. | The availability of flavored cigars/cigarillo, e-cigarettes or e-liquids. | In wave 1, pre-implementation, flavored product availability was 62.6%. At this time, product availability was 67.7% in control communities. Post-implementation, flavor product availability decreased by 27.2% in wave 1 communities but increased by 6.6% in control communities. In wave 2 communities, pre-implementation product availability was 79.8% and 75% in control communities at the same time. Post-implementation, availability dropped by 50.9% in wave two communities and decreased by 10.4% among control communities. In the period 6m-1y post-implementation 21 out of 38 communities with restrictions had 100% compliance. |
| Kingsley, M. 2019b (6) | Massachusetts | High school-aged youth | Pre and Post Analysis **(Compliance)** | 11 tobacco retailers and 593 students in Lowell; 47 tobacco retailers and 646 students Malden | Local restriction in Lowell on the sale of flavored tobacco to adult-only tobacco shops (2016) | Massachusetts Tobacco Control Program data on cigarette stock, and surveys of high school students using baseline and six-month follow-up. Lowell (restriction municipality) vs. Malden (no restriction). | The availability of flavored cigars/cigarillos, e-cigarettes or e-liquids. Youth access, current use, and ever use of flavored tobacco products. | Post-ban in Lowell, there was a significant reduction in the percentage of stores that sold flavored products (77.3% to 7.3%). In Malden, no significant change was observed (76.6% to 78.7%). There was a non-significant decrease in any flavored ever tobacco use in Lowell and a non-significant increase in Malden. The difference between the two communities was not statistically significant. There was a significant difference in current any flavored tobacco use between the two communities (-5.7%, p=0.03). However, the reduction in current any flavored tobacco use in Lowell was not significant ‒2.4 (‒6.2, 1.3) percentage points. |
| Pearlman, D. 2019 (7) | Providence, RI | High school-aged youth | Pre and Post Analysis **(Compliance)** |  | 2012 City-wide restriction on the sale of all flavored tobacco products excluding menthol to adult-only tobacco bars (enforced 2013) | Two rounds of store observation audits and 5 rounds of retail compliance checks. FDA compliance inspection data. 2012, 2016, and 2018 Providence Youth Experience Survey | The availability of flavored cigars/cigarillos, e-cigarettes or e-liquids. Youth access, current use, and ever use of flavored tobacco products. | Analysis of observational data showed that the availability of flavored products decreased from 37 of 90 stores in Round 1(41%) to 14 of 82 stores in Round 2 (17%). |

**References**

1. Brock B, Carlson SC, Leizinger A, D'Silva J, Matter CM, Schillo BA. A tale of two cities: exploring the retail impact of flavoured tobacco restrictions in the twin cities of Minneapolis and Saint Paul, Minnesota. Tob Control. 2019;28(2):176-80. Epub 2018/06/08. doi: 10.1136/tobaccocontrol-2017-054154. PubMed PMID: 29875154.

2. Farley SM, Johns M. New York City flavoured tobacco product sales ban evaluation. Tob Control. 2017;26(1):78-84. doi: 10.1136/tobaccocontrol-2015-052418. PubMed PMID: 26872486.

3. Farley SM, Schroth KR, Grimshaw V, Luo W, DeGagne JL, Tierney PA, Kim K, Pankow JF. Flavour chemicals in a sample of non-cigarette tobacco products without explicit flavour names sold in New York City in 2015. Tob Control. 2018;27(2):170-6. Epub 2017/04/13. doi: 10.1136/tobaccocontrol-2016-053552. PubMed PMID: 28400490; PMCID: PMC5870443.

4. Kephart L, Setodji C, Pane J, Shadel W, Song G, Robertson J, Harding N, Henley P, Ursprung WWS. Evaluating tobacco retailer experience and compliance with a flavoured tobacco product restriction in Boston, Massachusetts: impact on product availability, advertisement and consumer demand. Tob Control. 2019. Epub 2019/10/16. doi: 10.1136/tobaccocontrol-2019-055124. PubMed PMID: 31611423.

5. Kingsley M, Song G, Robertson J, Henley P, Ursprung WWS. Impact of flavoured tobacco restriction policies on flavoured product availability in Massachusetts. Tob Control. 2020;29(2):175-82. Epub 2019/02/20. doi: 10.1136/tobaccocontrol-2018-054703. PubMed PMID: 30777880.

6. Kingsley M, Setodji CM, Pane JD, Shadel WG, Song G, Robertson J, Kephart L, Henley P, Ursprung WWS. Short-Term Impact of a Flavored Tobacco Restriction: Changes in Youth Tobacco Use in a Massachusetts Community. Am J Prev Med. 2019;57(6):741-8. Epub 2019/11/02. doi: 10.1016/j.amepre.2019.07.024. PubMed PMID: 31668668.

7. Pearlman DN, Arnold JA, Guardino GA, Welsh EB. Advancing Tobacco Control Through Point of Sale Policies, Providence, Rhode Island. Preventing chronic disease. 2019;16:E129. Epub 2019/09/21. doi: 10.5888/pcd16.180614. PubMed PMID: 31538567; PMCID: PMC6795063.

8. Rogers T, Brown EM, McCrae TM, Gammon DG, Eggers ME, Watson K, Engstrom MC, Tworek C, Holder-Hayes E, Nonnemaker J. Compliance with a Sales Policy on Flavored Non-cigarette Tobacco Products. Tobacco regulatory science. 2017;3(2 Suppl 1):S84-s93. Epub 2017/01/01. doi: 10.18001/TRS.3.2(Suppl1).9. PubMed PMID: 30320155; PMCID: PMC6178822.

9. Rogers T, Feld A, Gammon DG, Coats EM, Brown EM, Olson LT, Nonnemaker JM, Engstrom M, McCrae T, Holder-Hayes E, Ross A, Boles Welsh E, Guardino G, Pearlman DN. Changes in cigar sales following implementation of a local policy restricting sales of flavoured non-cigarette tobacco products. Tob Control. 2019. Epub 2019/07/26. doi: 10.1136/tobaccocontrol-2019-055004. PubMed PMID: 31341001.
